# Gender Modifies the Association of Cognition With Age-Related Hearing Impairment in the Health and Retirement Study

**DOI:** 10.3389/fpubh.2021.751828

**Published:** 2021-12-17

**Authors:** Jing Yuan, Shuping Sang, Jessica Pham, Wei-Jia Kong

**Affiliations:** ^1^Department of Otorhinolaryngology, Union Hospital, Tongji Medical College, Huazhong University of Science and Technology, Wuhan, China; ^2^School of Medicine, Yunnan University, Kunming, China; ^3^School of Medicine, Case Western Reserve University, Cleveland, OH, United States; ^4^Institute of Otorhinolaryngology, Tongji Medical College, Huazhong University of Science and Technology, Wuhan, China

**Keywords:** aging, sex, gender, hearing impairment, cognitive impairment, risk factor, Health and Retirement Study (HRS)

## Abstract

**Introduction:** Despite growing recognition of hearing loss as a risk factor for late life cognitive disorders, sex and gender analysis of this association has been limited. Elucidating this is one means to advocate for holistic medicine by considering the psychosocial attributes of people. With a composite Gender Score (GS), we aimed to assess this among aging participants (50+) from the 2016 Health and Retirement Study (HRS) cohort.

**Methods:** The GS was derived from gender-related variables in HRS by factor analyses and logistic regression, ranging from 0 (toward masculinity) to 100 (toward femininity). GS tertiles were also used to indicate three gender types (GS tertile 1: lower GS indicates masculinity; GS tertile 2: middle GS indicates androgyny; GS tertile 3: higher GS indicates femininity). Univariate followed by multiple logistic regressions were used to estimate the Odds Ratio (OR) and 95% confidence intervals (CI) of cognitive impairment (assessed by adapted Telephone Interview for Cognitive Status) from hearing acuity, as well as to explore the interactions of sex and gender with hearing acuity. The risk of cognitive impairment among hearing-impaired participants was assessed using multivariable models including sex and gender as exposure variables.

**Results:** Five variables (taking risks, loneliness, housework, drinking, and depression) were retained to compute the GS for each participant. The distribution of GS between sexes partly overlapped. After adjusting for confounding factors, the OR for cognitive impairment associated with hearing impairment was significantly higher (OR = 1.65, 95% CI: 1.26, 2.15), and this association was not modified by female sex (OR = 0.77, 95% CI: 0.46, 1.27), but by androgynous gender (OR = 0.44, 95% CI: 0.24, 0.81). In the multivariable models for participants with hearing impairment, androgynous and feminine gender, as opposed to female sex, was associated with lower odds of cognitive impairment (OR of GS tertile 2 = 0.59, 95% CI: 0.41, 0.84; OR of GS tertile 3 = 0.60, 95% CI: 0.41, 0.87; OR of female sex = 0.78, 95% CI: 0.57, 1.08).

**Conclusions:** Hearing impairment was associated with cognitive impairment among older people, and this association may be attenuated by a more feminine GS.

## Introduction

The world's increasingly aging population has presented many common concomitants. Disabling hearing loss affects more than one-third of people over 65 across the globe, with its prevalence continuously increasing with age, yet it remains largely undertreated due to limited accessibility to hearing health care solutions for patients (1–5). Since the main complaint of age-related hearing impairment (ARHI) is difficulty following conversations in noisy environments, many patients regard it only a quality-of-life issue attendant on aging, and thus are reluctant to seek optimal medical evaluation and treatment, out of fear for stigmatization and high costs (3, 6). In truth, hearing loss can significantly affect the trajectory of healthy aging through precipitating physical and mental health outcomes, such as falls and disabilities, frailty, loneliness, depression, social isolation, and beyond (7–13). In particular, emerging evidence indicates a connection between ARHI and higher risk of cognitive decline in older people (14–19), and hearing loss has markedly been acknowledged as a major modifiable risk factor for dementia and Alzheimer's Disease (AD) (20).

Despite advancements in understanding the hearing-cognition link, challenges persist because there are many unknown aspects about this link. For example, it has already been known that ARHI and AD differ in prevalence in terms of sex—ARHI is more common and severe in men, while the incidence of AD is greater in women (21–23)—but whether sex predisposes older adults with hearing loss to greater risk of cognitive impairment is a subject of controversy. There is a lack of consensus regarding sex-differing risk in this area, when such data were disaggregated by biological sex (14, 24–26). Although social and cultural behavior has been one explanation for these previous findings, the systematic consideration of one's psychosocial sex—the state of being an older man or woman under the influence of culture and society—remains rare. In this regard, incorporating gender into analytic models is warranted to delineate the hearing-cognition link. Gender, as defined by the American Psychological Association (27), is related to the concept of sex (referring to the biological status indicated by, sex chromosomes, gonads, internal reproductive organs, and external genitals, etc.), but further reflects socially constructed identity, norms, attitudes, feelings and behaviors within a cultural context (28–31). Systematic reporting of sex and gender is increasingly recommended for its essential role in rigorous and accurate research, as medical research has historically centered on male subjects and physiology (32, 33). As for its relevance in our area of research, it has been reported that societal factors impact susceptibility to and prognosis of hearing loss and cognitive decline through entrenched sex roles and expectations (e.g., attitudes toward family and work, social networks, health-seeking behaviors, mental support, and coherence to hearing and cognitive rehabilitation) (3, 11, 20, 34, 35). Studies including gender-related variables could thus foster a more transparent and inclusive analysis with more information pertaining to sex, accounting for characteristic nuances on the gender spectrum, elucidating the psychosocial mechanism behind the contrasting risk of cognitive impairment between men and women from ARHI with other biological variables, and ultimately contributing to gender and social equity.

In the present study, we analyzed the core data from the 2016 Health and Retirement Study (HRS) (36). Although prior studies were carried out regarding the hearing-cognition association with the HRS cohort (16, 19, 37), few of them provided outcome data disaggregated by sex or gender. Our aim was to investigate how gender-related characteristics affect the hearing-cognition association in the older demographic alongside biological sex. The analysis herein estimated the risk for cognitive impairment of participants with and without hearing impairment, and the interaction of hearing status with sex and gender. We also looked into the association between sex, gender, and cognitive impairment in the hearing-impaired participants. To the best of our knowledge, this is the first study to explore the role of gender and sex in the relationship between late-life hearing impairment and cognitive function.

## Materials and Methods

### Study Participants

Participants were enrolled in HRS, a biennial longitudinal survey in the United States, designed to provide nationally representative, population-based content on the health, economics and demographic aspects of aging for researchers (36). The analytic sample of the present study was from the 2016 HRS core interview, including information about the respondents' demographic characteristics, general mental and physical health, family, work and retirement life, psychosocial factors, as well as cognitive functioning. Surveys and cognitive assessment were conducted in English or Spanish, as the participant preferred. To fall within the scope of the present study, participants who underwent objective hearing testing were selected as the analytic sample. As HRS assigns half of the core sample for physical measures (including objective hearing testing since 2016) at each wave (38), this means that missing data here were partly due to the sampling strategy. The HRS protocols were reviewed and approved by the Institutional Review Board of the University of Michigan. Participants provided their written informed consent to participate before the HRS interview (39).

**Figure 1:**
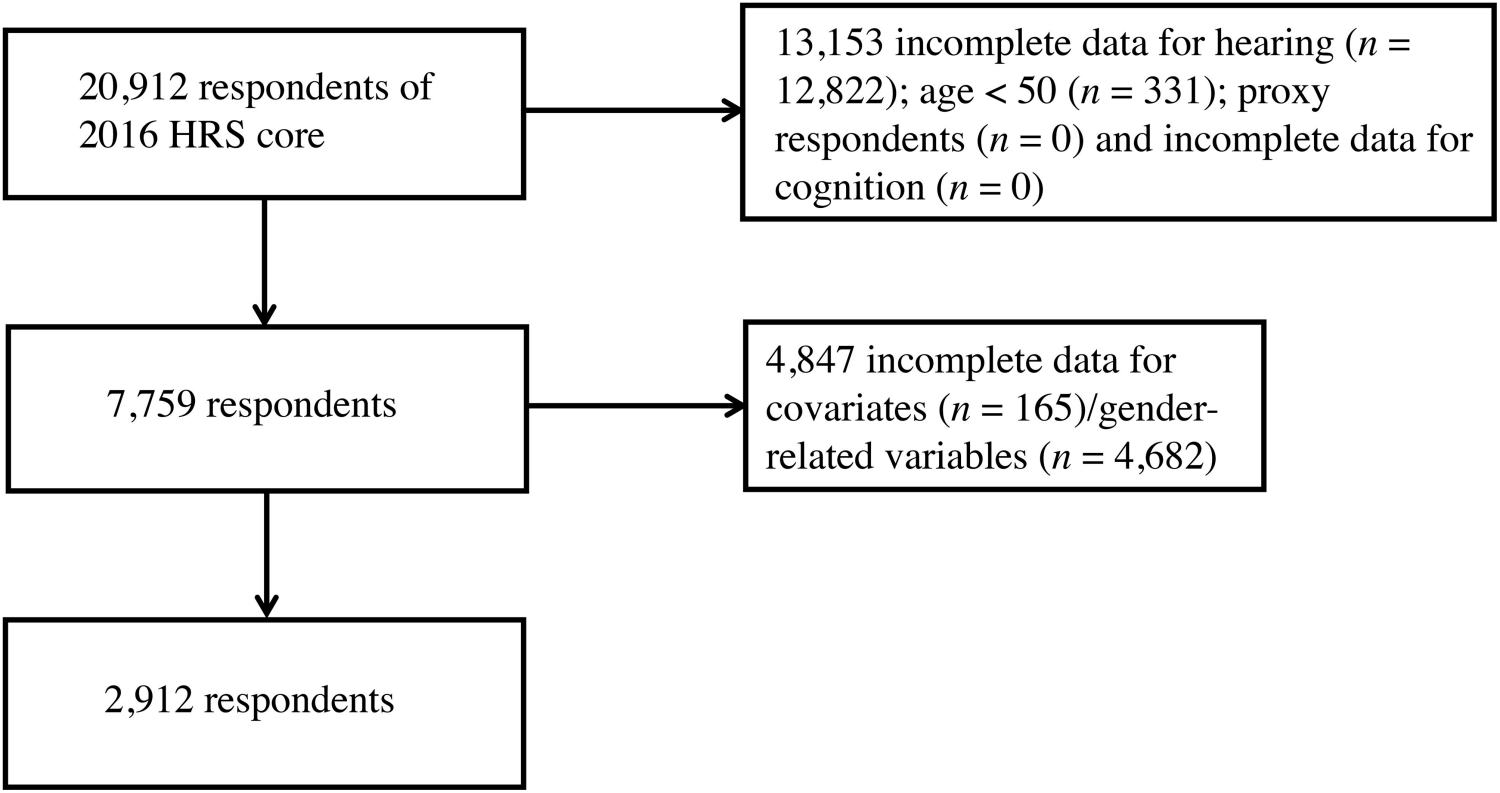
Flow diagram of subject selection from the 2016 HRS core. HRS, Health and Retirement Study.

Of the 2016 HRS core sample of 20,912 respondents, 18,000 persons were excluded for being a proxy interviewee or below the age of 50, incomplete hearing measure data, incomplete cognitive testing results and missing covariates and gender-related variables, leaving a sample of 2,912 for the final analyses (Figure 1). Basic characteristics of participants who were excluded were compared with the analytic sample in Supplementary Table 1.

### Hearing Measures

The hearing status of HRS participants was evaluated by a hearing screening test, which was administered by trained HRS staff using the HearCheck Screener^®^ (Siemens, Germany) in a quiet ambience (40, 41). Six acoustic signals (55 decibels in hearing level (dB HL), 35 dB HL and 20 dB HL at a mid-frequency of 1 kHz; 75 dB HL, 55 dB HL and 35 dB HL at a high frequency of 3 kHz) were played on one unaided ear, then the number of tones heard in the test was counted and recorded. The same procedure next was applied on the other ear. Hearing fewer than six tones in the best hearing ear was evaluated as having hearing impairment (41, 42).

### Cognitive Measures

Cognitive functioning in HRS was assessed from a battery adapted from the Telephone Interview for Cognitive Status (43). It includes items to test the participants' cognitive ability of episodic memory (immediate and delayed word recall; respondents were asked to recall 10 unrelated nouns the interviewer just read to them, and again, after a delay, yielding a score of 0 to 20), working memory (serial 7's test, respondents were asked to subtract 7 from 100 and continue subtracting 7 for five times in total, yielding a score of 0 to 5), attention and processing speed (counting backwards, respondents were asked to count back from 20 for 10 continuous numbers for two trials, yielding a score of 0 to 2). The overall cognitive performance was measured as summing scores of the above items (ranging from 0 to 27), with higher scoring representing better cognition of the participant. We classified cognitive performance of respondents into two categories: normal cognition (scored 12 to 17) and cognitive impairment (scored 0 to 11) in reference to others (44–46).

### Gender Score (GS) Construction

**Figure 2:**
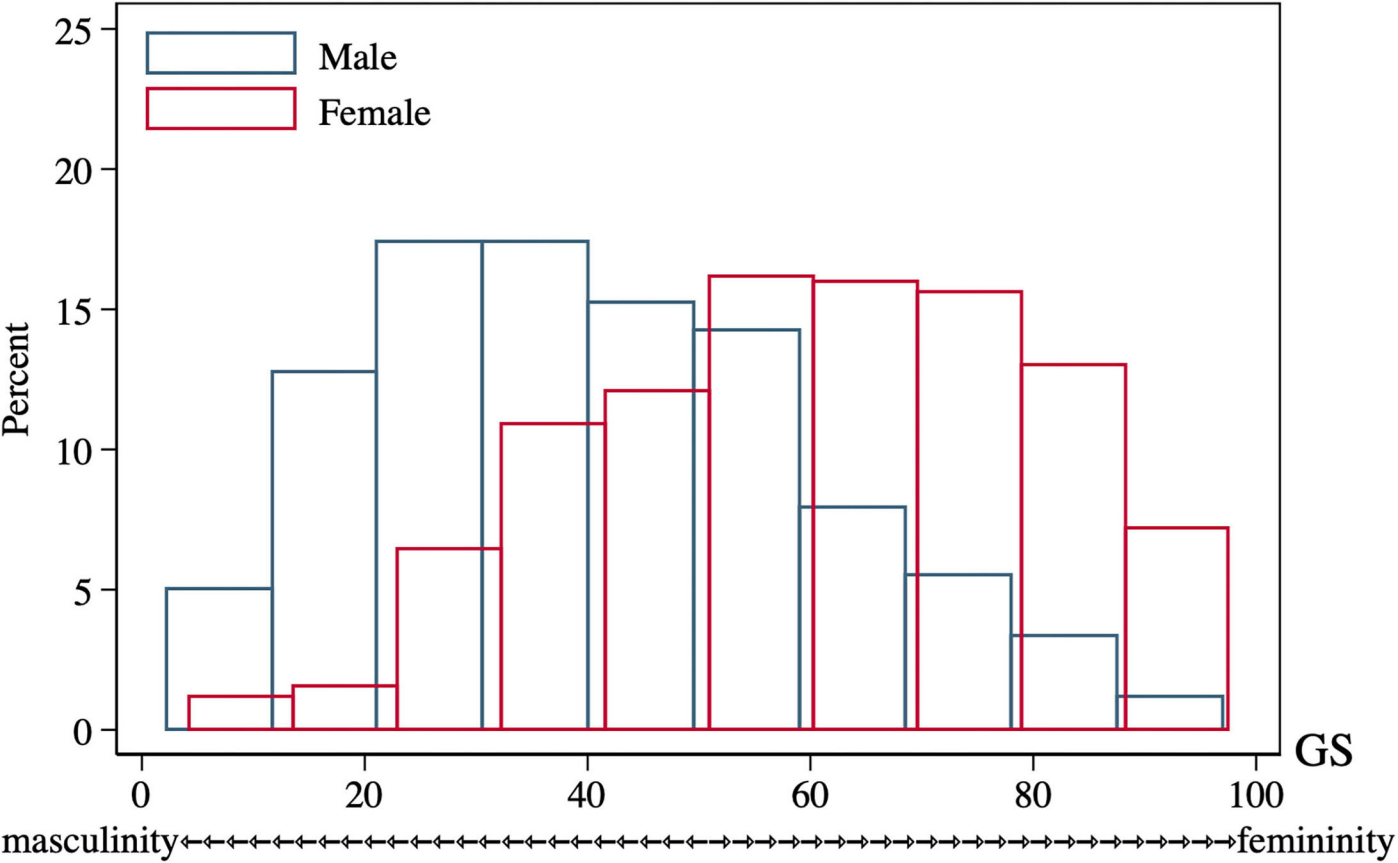
GS distribution of 2016 HRS participants of both sexes (*n* = 2,912). GS, Gender Score; HRS, Health and Retirement Study.

To our knowledge, no gender measures had been used with the HRS participants, so the Gender Score for the current study was constructed according to previous protocols from other cohorts (30, 47–49). Firstly, variables deemed as gender-related were selected from the 2016 HRS core datasets. Two investigators (J.Y. and S.S.) independently screened the HRS core variables and made the selection in concert with the four aspects of a gender construct: gender roles, gender identity, gender relationships, and institutionalized gender (28), as well as with the recommended process for analyzing gender (31). Discrepancies were resolved with the whole author group before identifying 13 gender-related variables (Supplementary Table 2). Next, a factor analysis with the principal-component factor method was performed to reduce the dimensionality of these initial variables. Factors with an eigenvalue >1 were retained for a varimax orthogonal rotation to further increase interpretability (see original factor loadings in Supplementary Table 3). Variables with a factor loading of 0.40 or greater for one factor and lower for the remaining factors, as well as a communality >0.40 were retained (Supplementary Table 4). This was followed by a logistic regression to identify the relationship between these retained variables (independent variables) and biological sex (dependent variable: female = 1, male = 0), determining which of them were associated with the reality of belonging to a given sex group. Variables not significantly associated with biological sex were omitted from the regression model sequentially until all remaining variables had *P*-values below 0.05 (Table 1). Lastly, a GS from the propensity score of the logistic regression was computed, estimating the conditional probability of being female in the model when considering these gender-related variables as confounders. The receiver operator characteristic (ROC) curve of GS compared to sex is graphed in Supplementary Figure 1; the area under the curve (AUC) and its 95% confidence interval (CI) was estimated. The GS for each participant ranges from 0 (toward masculine characteristics) to 100 (toward feminine characteristics), and the distribution of GS of HRS participants by sex is presented in Figure 2.

**Table 1:** Gender-related variables for the construction of GS, *n* = 2,912.

**Variables**	**Coefficient (95% CI)**	***P-*value**
Risk willingness	−0.23 (−0.26, −0.19)	**<0.001**
Loneliness	−0.57 (−0.78, −0.36)	**<0.001**
Less participation in household tasks	−0.88 (−0.98, −0.79)	**<0.001**
Regular drinking	−0.50 (−0.76, −0.24)	**<0.001**
Depression	0.15 (0.10, 0.21)	**<0.001**

*CI, Confidence Interval; GS, Gender Score. Bold values are reached the significant level of P < 0.05*.

### Covariates

Variables in the HRS cohort that could modify the hearing-cognition link were derived as covariates here, including age (years), sex (male; female), GS tertile (three categories from GS, GS tertile 1: lower GS indicative of masculine type; GS tertile 2: middle GS indicative of androgynous type; GS tertile 3: higher GS indicative of feminine type), ethnicity (White; Black; others), educational attainment (lower than high school; high school or equivalent; college and above), marital status (married; spouse absent, separated, divorced, or widowed; never married), smoking (current smoker; not a current smoker), exercise (at least one vigorous activity per week or not), cardiovascular and cerebrovascular disease [CVD, as a 0–5 composite score to sum the presence of diagnosed heart problems, hypertension, stroke, and diabetes (17)].

### Statistic Methods

We compared the characteristics of HRS participants across sex and gender tertile using Chi-square tests for categorical variables and Students' *t*-tests for continuous variables. When the data of specific variables were not assumed as normal and had unequal variances according to the Shapiro-Francia test and Bartlett's test, non-parametric Mann-Whitney U-tests and Kruskal-Wallis tests were used to compare differences between sex and gender. Box plots were graphed for variables with the same median.

Univariate followed by multiple logistic regression models were applied to examine the cross-sectional association between hearing acuity (independent variable) and the risk of cognitive impairment (dependent variable) in this study, as well as to explore the interactions of sex and gender with hearing acuity. Multivariable regression was performed in four models with the above covariates included (Model I: adjusted for age and sex; Model II: adjusted for age, sex, GS tertile, education, ethnicity, marital status, CVD, smoking, physical activities; Model III: adjusted for model II and an interaction term of hearing × sex; Model IV: adjusted for model II and an interaction term of hearing × GS tertile). Odds Ratios (OR) and their 95% CI were reported. To determine the relative contributions of sex and gender, we used multiple logistic regressions to evaluate the risk of cognitive impairment among hearing-impaired participants including sex and gender as exposure variables. Odds Ratios and their 95% CI and c statistics were computed for three models (Model I: included age, sex, education, ethnicity, marital status, CVD, smoking, and physical activities as covariates; Model II included age, GS tertile, education, ethnicity, marital status, CVD, smoking, and physical activities as covariates; Model III included age, sex, GS tertile, education, ethnicity, marital status, CVD, smoking, and physical activities as covariates).

The statistical analyses were performed by Stata 14.1 MP (Stata Corp, College Station, TX, US). Statistical significance was considered at a *P*-value < 0.05, unless otherwise indicated.

## Results

### Participants' Characteristics

There were 20,912 respondents that underwent the 2016 HRS core tests and interviews. In the selecting process (Figure 1), participants were excluded for incomplete data for hearing (*n* = 12,822), age below 50 (*n* = 331), being proxy respondents (*n* = 0), missing data for cognition evaluations (*n* = 0), incomplete data for covariates (*n* = 165) and missing data to measure gender (*n* = 4,682). The characteristics of participants by sex are presented in Table 2. Of these, 50.7% were female (*n* = 1,476). On average, female subjects seemed to be younger, living without spouses, having fewer CVD, and more likely to have depressive feelings. In contrast, a higher proportion of male subjects were married, sustaining more CVD risks, leading an active lifestyle, but also in the habit of regular drinking and feeling lonely. There were no statistical differences in ethnicity or education attainment across the two sex groups. As for variables of integer values that presented with the same median (CVD, cognitive testing score and depression scale score), their difference was further compared with box plots (Supplementary Figure 2).

**Table 2:** Characteristics of participants by sex, HRS 2016, *n* = 2,912.

**Characteristics**	**Male *n* = 1,436**	**Female *n* = 1,476**	***P*-value**
Median age, years (IQR)	65 (17)	63 (15)	**<0.001**
Gender			**<0.001**
GS tertile 1	730 (50.84)	241 (16.33)	
GS tertile 2	479 (33.36)	491 (33.27)	
GS tertile 3	227 (15.81)	744 (50.41)	
Ethnicity			0.309
White	1,112 (77.44)	1,135 (76.90)	
Black	183 (12.74)	212 (14.36)	
Other	141 (9.82)	129 (8.74)	
Marital status			**<0.001**
Never married	34 (2.37)	38 (2.57)	
Spouse absent	147 (10.24)	243 (16.46)	
Married	1,255 (87.40)	1,195 (80.96)	
Education			0.173
Less than high school	157 (10.93)	152 (10.30)	
High school or equivalence	438 (30.50)	498 (33.74)	
Some college and above	841 (58.57)	826 (55.96)	
Median CVD score (IQR)	1 (3)	1 (2)	**<0.001**
≥1 CVD	1,026 (71.45)	941 (63.75)	**<0.001**
Current smoker	150 (10.45)	158 (10.70)	0.820
Regular drinker	201 (14.00)	125 (8.47)	**<0.001**
Regular vigorous physical activity	664 (46.24)	493 (33.40)	**<0.001**
Hearing impairment	806 (56.13)	734 (49.73)	**0.001**
Cognitive testing score (IQR)	16 (5)	16 (5)	**<0.001**
Cognitive impairment	231 (16.09)	182 (12.33)	**0.004**
Median CESD score (IQR)	0 (1)	0 (2)	**<0.001**
≥1 negative feeling in CESD	599 (41.71)	727 (49.25)	**<0.001**
Median loneliness score (IQR)	1.45 (0.64)	1.36 (0.64)	**<0.001**

*Data are reported as n (%) unless otherwise specified. Bold values: reached the significant level of P < 0.05. CESD, Center for Epidemiologic Studies Depression Scale; CVD, cardiovascular disease; GS, Gender Score; HRS, Health and Retirement Study; IQR, interquartile range*.

GS, which estimated the conditional probability of being female, was computed for each participant with five variables related to gender (Table 1). They were “willing to take risks,” “loneliness,” “reluctance to undertake household chores,” “regular drinking,” and “depression,” which yielded an AUC of 0.7586 (95% CI: 0.7413, 0.7758) to separate the two sexes (Supplementary Figure 1). Considerable overlap existed on the GS spectrum between sexes (Figure 2), and both sex groups incorporated subjects evaluated with GS values of the opposite expected gender (males: 33.4% had an androgynous gender type and 15.8% had a feminine gender type; females: 33.3% had an androgynous gender type and 16.3% had a masculine gender type, Table 2). After comparing participants by gender tertile, included characteristics were significantly different across gender types except for the incidence of hearing and cognitive impairment (Table 3). In comparison, hearing impairment and cognitive impairment were more prevalent in the population with male sex (Table 2). For variables of integer values that presented with the same median (CVD, cognitive testing score and depression scale score), their difference was also compared with box plots (Supplementary Figure 3).

**Table 3:** Characteristics of participants by gender, HRS 2016, *n* = 2,912.

**Characteristics**	**GS tertile 1 *n* = 971**	**GS tertile 2 *n* = 970**	**GS tertile 3 *n* = 971**	***P-*value**
Median age, years (IQR)	64 (16)	63 (16)	65 (16)	**0.0405**
Sex, female	241 (24.82)	491 (50.62)	744 (76.62)	**<0.001**
Ethnicity				**<0.001**
White	784 (80.74)	738 (76.08)	725 (74.67)	
Black	99 (10.20)	121 (12.47)	175 (18.02)	
Other	88 (9.06)	111 (11.44)	71 (7.31)	
Marital status				**<0.001**
Never married	18 (1.85)	15 (1.55)	39 (4.02)	
Spouse absent	78 (8.03)	94 (9.69)	218 (22.45)	
Married	875 (90.11)	861 (88.76)	714 (73.53)	
Education				**0.003**
Less than high school	113 (11.64)	99 (10.21)	97 (9.99)	
High school or equivalence	293 (30.18)	285 (29.38)	358 (36.87)	
Some college and above	565 (58.19)	586 (60.41)	516 (53.14)	
Median CVD score (IQR)	1 (2)	1 (2)	1 (2)	**0.0033**
≥1 CVD	660 (67.97)	617 (63.61)	690 (71.06)	**0.002**
Current smoker	101 (10.40)	80 (8.25)	127 (13.08)	**0.002**
Regular drinker	176 (18.13)	104 (10.72)	46 (4.74)	**<0.001**
Regular vigorous physical activity	456 (46.96)	385 (39.69)	316 (32.54)	**<0.001**
Hearing impairment	515 (53.04)	492 (50.72)	533 (54.89)	0.183
Cognitive testing score (IQR)	16 (5)	16 (6)	16 (4)	**0.0261**
Cognitive impairment	158 (16.27)	123 (12.68)	132 (13.59)	0.062
Median CESD score (IQR)	0 (1)	0 (1)	1 (2)	**<0.001**
≥1 negative feeling in CESD	379 (39.03)	421 (43.40)	526 (54.17)	**<0.001**
Median loneliness score (IQR)	1.45 (0.73)	1.36 (0.64)	1.36 (0.64)	**<0.001**

*Data are reported as n (%) unless otherwise specified. Bold values: reached the significant level of P < 0.05. CESD, Center for Epidemiologic Studies Depression Scale; CVD, cardiovascular disease; GS, Gender Score; HRS, Health and Retirement Study; IQR, interquartile range*.

### Cognitive Performance With Age and Hearing Acuity

**Figure 3:**
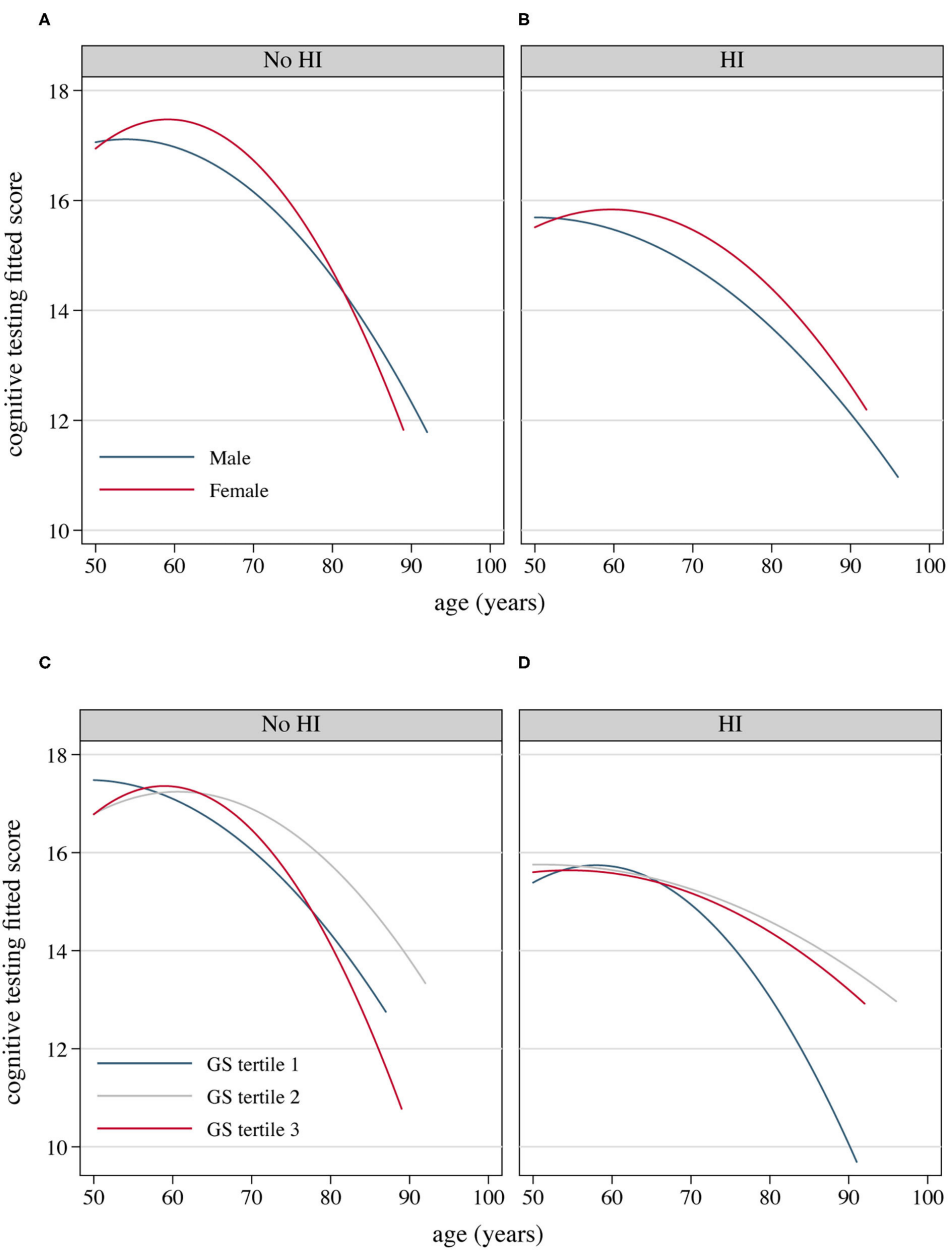
Fitted cognitive testing scores as the increase of the age of participants, by hearing acuity, sex and gender, 2016 HRS (*n* = 2,912). **(A)** Fitted cognitive decline as the age of participants with normal hearing increases by sex; **(B)** Fitted cognitive decline as the age of HRS participants with hearing impairment increases by sex; **(C)** Fitted cognitive decline as the age of HRS participants with normal hearing increases by GS tertile; **(D)** Fitted cognitive decline as the age of HRS participants with hearing impairment increases by GS tertile. GS, Gender Score; HI, hearing impairment; HRS, Health and Retirement Study.

**Figure 4:**
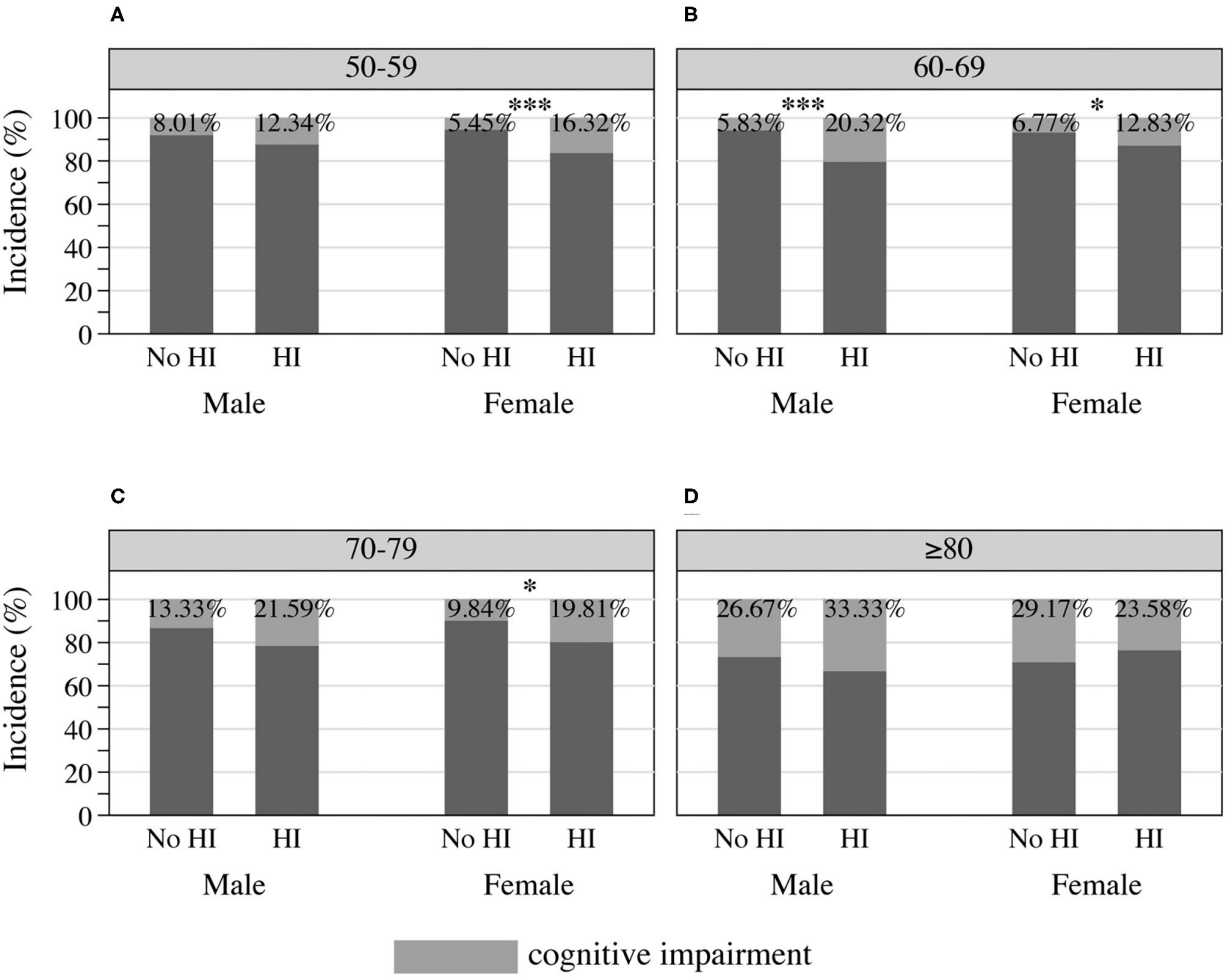
Incidence of cognitive impairment among HRS participants by sex and hearing acuity at the age of **(A)** 50–59, **(B)** 60–69, **(C)** 70–79, and **(D)** 80 or older, 2016 HRS (*n* = 2,912). *P < 0.05, ***P < 0.001. HI, hearing impairment; HRS, Health and Retirement Study.

**Figure 5:**
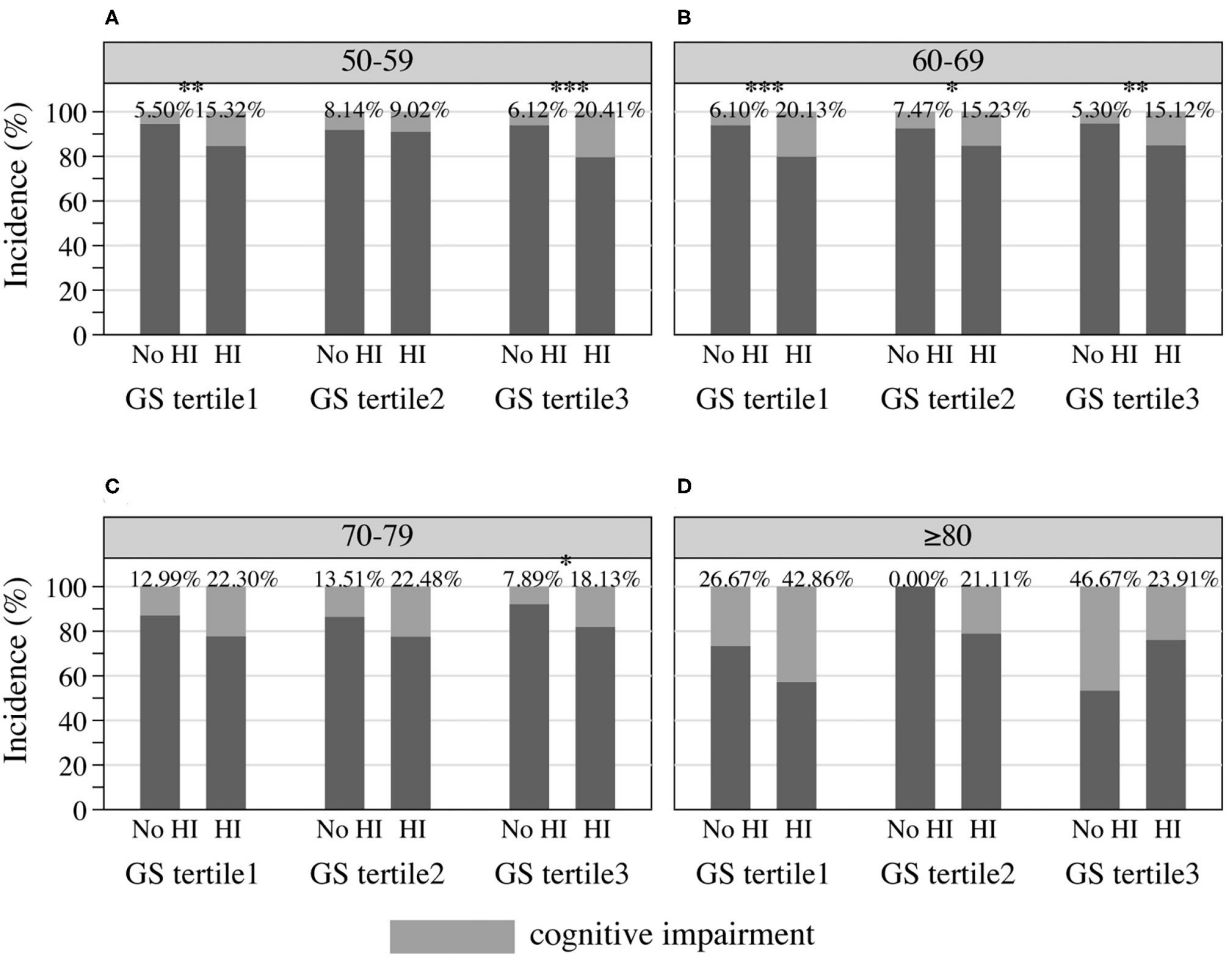
Incidence of cognitive impairment among HRS participants by gender and hearing acuity at the age of **(A)** 50–59, **(B)** 60–69, **(C)** 70–79, and **(D)** 80 or older, 2016 HRS (*n* = 2,912). *P < 0.05, **P < 0.01, ***P < 0.001. HI, hearing impairment; HRS, Health and Retirement Study; GS, Gender Score.

Cognitive performance, based on scores in the HRS core cognitive testing, declined as the overall age of participants increased (Figure 3). Participants with hearing impairment scored lower in cognition than those with normal hearing acuity of the same age. Male sex participants showed poorer cognitive performance compared to females (Figure 3B), and similarly, people presenting with masculine characteristics showed poorer cognitive performance in contrast to those of androgynous or feminine types of gender (Figure 3D). The estimated incidence of cognitive impairment, with and without hearing impairment, is presented in Figure 4 (by sex and age) and Figure 5 (by gender and age). The incidence of cognitive impairment was higher for male sex participants with hearing impairment (20.32%) than for those without hearing impairment of the same sex (5.83%) in the 60–69 age group (Figure 4B). Among female participants in the age groups of 50–59, 60–69, and 70–79, the incidence of cognitive impairment was 16.32, 12.83, and 19.81% for those whose hearing acuity was impaired, and was 5.45, 6.77, and 9.84% for those whose hearing acuity was intact, respectively (Figures 4A–C). The incidence of cognitive impairment for participants of the middle GS tertile was higher for those with hearing impairment (15.23%) than for those without hearing impairment (7.47%) in the age group of 60–69 years (Figure 5B). Among those evaluated with the masculine and feminine gender type, the incidence of cognitive impairment was significantly higher among the hearing-impaired participants compared to the cognitive impairment incidence of those of normal hearing, except for participants aged 80 and above (age 50–59: 15.32 vs. 5.50% among masculine gender participants, 20.41 vs. 6.12% among feminine gender participants, Figure 5A; age 60–69: 20.13 vs. 6.10% among masculine gender participants, 15.12 vs. 5.30% among feminine gender participants, Figure 5B; age 70–79: 18.13 vs. 7.89% among feminine gender participants, Figure 5C).

### Association Between Hearing Impairment and Cognitive Impairment

Multivariable odds for cognitive impairment from hearing impairment are presented in Table 4. In the crude model without accounting for covariates, hearing impairment was associated with higher odds of cognitive impairment compared with normal hearing (OR = 2.85, 95% CI: 2.26, 3.60). After controlling for age and sex, the association remained significant (OR = 2.23, 95% CI: 1.75, 2.85, Model I). The significant association of cognitive impairment with hearing impairment remained, albeit attenuated, with adjustment for gender, demographic and other health factors (OR = 1.65, 95% CI: 1.26, 2.15, Model II). Two interaction terms were introduced in Model III and Model IV, respectively. The association was not affected by female sex (OR = 0.77, 95% CI = 0.46, 1.27), but attenuated by the second GS tertile (OR = 0.44, 95% CI = 0.24, 0.81).

**Table 4:** Association of hearing impairment with cognitive impairment, HRS 2016, *n* = 2,912.

	**Odds ratio (95% CI)**				
	**Model crude^a^**	**Model I^b^**	**Model II^c^**	**Model III^d^**	**Model IV^e^**
HI, *n* = 1,540	**2.85 (2.26, 3.60)**	**2.23 (1.75, 2.85)**	**1.65 (1.26, 2.15)**	**1.88 (1.30, 2.70)**	**2.47 (1.59, 3.86)**
HI*female sex				0.77 (0.46, 1.27)	
HI*GS tertile 2					**0.44 (0.24, 0.81)**
HI*GS tertile 3					0.64 (0.34, 1.21)

a *Unadjusted,*

b *adjusted for age and sex,*

c *adjusted for age, sex, GS tertile, education, ethnicity, marital status, CVD, smoking, physical activities,*

d *adjusted for model II and the interaction term of sex and hearing*.

e *Adjusted for model II and the interaction term of gender and hearing. Bold values: reached the significant level of P < 0.05. CI, Confidence Interval; CVD, cardiovascular disease, GS, Gender Score; HI, hearing impairment; HRS, Health and Retirement Study*.

### Association Between Sex, Gender, and Cognitive Impairment Among the Participants With Hearing Impairment

Table 5 shows that, in multiple regression models, including either sex or gender as the exposure variables, female sex was associated with a decreased risk of cognitive impairment among participants with hearing impairment (OR = 0.66, 95% CI = 0.49, 0.88, Model I), so was the second and third GS tertiles (OR of GS tertile 2 = 0.56, 95% CI = 0.39, 0.79; OR of GS tertile 3 = 0.53, 95% CI = 0.37, 0.75, Model II). When taking account of both sex and gender, the second and the third GS tertiles, as opposed to female sex, were associated with higher odds of cognitive impairment among those with hearing impairment (OR of GS tertile 2 = 0.59, 95% CI = 0.41, 0.84; OR of GS tertile 3 = 0.60, 95% CI = 0.41, 0.87; OR of female sex = 0.78, 95% CI = 0.57, 1.08, Model III). The c statistics suggest better predictive accuracy of cognitive outcome when including GS in the analytic models.

**Table 5:** Multivariable-adjusted Odds Ratio for cognitive impairment among participants with hearing impairment, HRS 2016, *n* = 1,540.

	**Exposure variables**	**Odds Ratio (95% CI)**	**C statistic**
Model I^a^	Female sex	**0.66 (0.49, 0.88)**	0.7772
Model II^b^			0.7816
	GS tertile 2	**0.56 (0.39, 0.79)**	
	GS tertile 3	**0.53 (0.37, 0.75)**	
Model III^c^			0.7829
	Female sex	0.78 (0.57, 1.08)	
	GS tertile 2	**0.59 (0.41, 0.84)**	
	GS tertile 3	**0.60 (0.41, 0.87)**	

a *Adjusted for age, sex, education, ethnicity, marital status, CVD, smoking, physical activities,*

b *adjusted for age, GS tertile, education, ethnicity, marital status, CVD, smoking, physical activities,*

c *adjusted for age, sex, GS tertile, education, ethnicity, marital status, CVD, smoking, physical activities. Bold values: reached the significant level of P < 0.05. CI, Confidence Interval; CVD, cardiovascular disease; GS, Gender Score*.

## Discussion

By developing an approach to measure the gender of participants in the 2016 HRS cohort, we found that hearing impairment was associated with an increased risk for cognitive impairment among older adults. Those who exhibited masculine characteristics were at greater risk of cognitive impairment from impaired hearing function, compared to those who exhibited androgynous or feminine traits. Incorporation of gender in the analytic models increased predictive accuracy, and the risk for cognitive impairment seemed to be attenuated by a more feminine GS, independent of female sex. This suggests that gender does have an impact on the hearing-cognition association. To our knowledge, the study provides the first proof of the effect of gender (the social construct of sex that reflects social roles, behavior patterns and life context) on the association between hearing acuity and cognitive impairment in late life. We have added to the evidence that hearing impairment is a risk factor for cognitive disorders among older persons with a composite score of gender and extended the psychosocial aspect of sex in shaping cognitive outcomes.

The sex-specific association between hearing loss and cognition impairment has previously been documented. There were studies that evaluated the sex-specific risk for cognitive impairment for older hearing-impaired people, either by analyzing prospective single-sex cohorts, mixed cohorts, or cross-sectional populations (14, 24–26, 50–52). Among the existing evidence, some did not find the association between hearing and cognition differing by sex (14, 50), while some reported impaired hearing posed a higher risk for cognitive decline in a specific sex group (25, 51, 52). We postulate that in these controversial findings, there may be confounding factors beyond sex dichotomy. First, much information would be missed if investigators did not consider potential gender-related variables, leading to restricted exploration of the psychosocial influence experienced by the participants. Secondly, in addition to our study, prior studies have suggested that Gender Score is not normally distributed between the two sex groups, and partly overlaps (47, 49), indicating that gender characteristics exist on a continuum that necessitates sophisticated measures to account for its nuances. Indeed, our data suggest that there were differences between GS tertiles that did not otherwise show by binary sex groups. Also, the association of hearing and cognition was not modified by sex, but by gender, emphasizing that the cognitive outcomes from hearing functioning during the aging process could be better explained in a gender-sensitive manner.

The fluidity of gender is also reflected in a temporal manner: our findings confirm that the main gender influences experienced by older participants were mental status and family obligations, while for working cohorts, gender was influenced by career issues (47, 53). This was also consistent with conclusions of another older, non-working cohort (48). A number of environmental and socioeconomic transitions may disrupt retiring populations' health status (35). Gender could therefore be a good candidate to reflect the contextual change in terms of time and place. Following previous arguments (48, 49), we also identified the predictive role of gender in cognitive impairment, independent of sex. This indicates that prevention of cognitive decline among older adults with hearing problems could benefit from gender-based health assessments. By complementing the social, cultural, and psychological dimensions of sex, our analysis recognizes the gendered pattern of propensity for cognitive impairment with aging, and emphasizes the necessity of integrating gender into health research.

Our findings may help explain the psychosocial mechanisms behind the risk of cognitive impairment between older men and women with hearing problems. Biological infliction and behaviors under social circumstances both gave rise to hearing and cognitive problems over time (41, 54, 55). This is the area where gender can play a role, because it includes the psychosocial and behavioral components in addition to sex (28). To illustrate this in the hearing-cognition context: hearing-impaired older individuals may choose to cut off activities involving hearing and talking to avoid stigma; as such, they are more likely to spend working and private time alone (56). Unfortunately, withdrawal from social contexts and gradual loss of connectedness would further lead to faster development of cognitive decline for the older persons (57). There was also a hypothesis that one of the causes of cognitive decline and dementia is social isolation (20, 40). From this point to our results, our construction of GS shows that experiencing strong feelings of loneliness [derived from *feeling lack companionship, left out, isolated from others, not “in tune” with the people around, alone, there are no people I can talk to, there are no people I can turn to, there are no people who really understand me, there are no people I feel close to, not part of a group of friends, do not have a lot in common with the people around* (58, 59)] is closely related to the masculine type of gender (Table 1). This probably explains why we observed that persons with hearing impairment and lower GS (socially ascribed to masculinity) had greater odds of cognitive impairment, compared to their counterparts of higher GS (socially ascribed to femininity) here (Tables 4, 5). Contrary to the current research, other investigations (11, 35) have concluded that aged women tend to be more likely to suffer from social isolation due to hearing loss, and provided explanations from the perspective of gender-specific roles and social conventions. So, this rationale in our study remains to be fully elucidated with more evidence. Additionally, the findings here show that participants with masculine characteristics were associated with less participation in the household tasks, which is a major indicator of cognitive decline and clinical dementia (60). But we are not sure whether declined cognition prevented these participants from fulfilling domestic chores, or whether it was their masculine traits and difficult hearing that made them reluctant to undertake such tasks. It is necessary to verify the novelty here, since domestic workload has been reported to be associated with health outcomes such as coronary heart diseases (49).

Our results should be interpreted considering some limitations. The sex and gender analysis here was based on information from the HRS interviews. The sex variable was obtained through the respondents' answers, so it could be contradictory from the biological indicators of sex, based on some respondents' point of view. This answer could not provide information as to whether the interviewee had received any surgeries or treatment for the variations of internal and/or external genitals. Similarly, the Gender Score we used here was devised according to what were available in the HRS psychosocial questionnaires (59), compromising the validity and generalizability of the measure to apply to other contexts, although the ROC curve shows fair-to-good sensitivity/specificity to separate the two biological sexes. Also, the older participants we selected for analysis were different from the excluded participants in many aspects (Supplementary Table 1), which indicates that the missing data may not be random. To some extent, this is because, in the 2016 wave, HRS chose half of the core sample to assign to the face-to-face interview, enhanced with physical measures and following questionnaires (e.g., hearing testing and psychosocial measures), leaving large portions of missing data from the full core dataset. Still, excluding participants who did not meet the criteria of the present study could weaken estimation of the association between hearing impairment and cognitive impairment. Furthermore, no dose-response effect was confirmed in either the association of hearing impairment with cognitive impairment or the risk for cognitive impairment among participants with hearing impairment, making the use of Gender Score to estimate the chance of cognitive impairment debatable, although the c-statistics showed the inclusion of gender increased some accuracy of prediction. Even though this pragmatic approach to measure gender has been used in different cohorts (48, 49, 61), we predict with caution that the influence of gender on cognition outcomes observed here will be repeated precisely in other older populations, until widely tested and recognized measures of gender are introduced in the future. The HRS cognitive testing here is largely instructed aurally, depending on sufficient hearing function to understand the task, which means that the results of the cognitive testing may be somewhat confounded due to hearing impairment (62, 63). This should be given due caution because evidence found that neuropsychological assessments of some cognitive domains, which are supposed to reflect cognitive dysfunction, can be confounded by degraded hearing, including decreased central hearing (64, 65). The absence of clinical evaluations on cognition and objective information on peripheral and central hearing capacity in the present study leaves room for bias—the differential diagnosis of other types of dementia cannot be ascertained since performance from neuropsychological instruments is not equivalent to a stringent dementia diagnosis; substandard audiometric assessment may overestimate stronger risk of cognitive decline (66, 67); the contribution of central auditory deficits to cognitive impairment is still unknown. We will pay close attention to the validation of our results by newly released datasets, especially from cohorts enhanced with comprehensive cognitive assessment [e.g., a subset of the HRS, the Harmonized Cognitive Assessment Protocol (HCAP) Project (68)]. Finally, we are unable to determine the temporal precedence of hearing impairment and subsequent cognitive decline because of the cross-sectional design of this study, and recalling bias of variables (e.g., social activities, lifestyles) and the lack of critical information (e.g., brain injury history, occupational and recreational noise exposure, ototoxic medications)—all of which could potentially bring about changes to our analytic models.

There is a need for longitudinal analyses to validate the gender interaction in the hearing-cognition association, if any, to search for answers regarding how gender-related characteristics change and how this change influences the association over time. Multi-level studies have been initiated to examine the shared pathology of hearing and cognition from animal models and cell lines. It would be informative to continue to apply the gender-related social factors that we identified in the current study to basic research, producing more rigorous results to increase our knowledge of the origins of gendered behavior analogous to human experiences (22, 69).

Taken together, our findings reveal that, by using a pragmatic portrayal of gender, gender-related factors contribute to cognitive health among the hearing-impaired older persons. Cognitive impairment associated with hearing loss may be attenuated by more feminine characteristics rather than female sex, and this is possibly related to their gender-specific health status and habits. These preliminary results may help researchers and physicians to gain insight into how gender is involved in geriatric conditions and encourage them to probe on gender factors, which may affect health outcomes in relation to their own work. The results should prompt physicians to be cognizant of whether behaviors and social circles of their hearing-impaired patients could affect cognitive outcomes, and to take proper measures to consider gender-diverse people, at different life stages, as they possibly experience different health risks. The results indicate that prevention of cognitive decline among older adults with hearing problems could benefit from gender-based health assessments.

This may also inspire developing strategies and prevention campaigns to address inequity and to cater for individuals of specific gender needs. There is still a lot of paucity in this critically under-researched area and more studies are needed to be done to guarantee thorough, beneficial health strategies for the aging population.

## Data Availability Statement

Publicly available datasets were analyzed in this study. This data can be found at: https://hrs.isr.umich.edu/about.

## Ethics Statement

The studies involving human participants were reviewed and approved by the Institutional Review Board of the University of Michigan. The patients/participants provided their written informed consent to participate in this study.

## Author Contributions

W-JK and JY conceived and designed the work. JY and JP prepared the manuscript. JY, W-JK, and SS conducted the data analysis. JY and SS prepared figures and tables. All authors reviewed and approved the manuscript.

## Funding

This work was supported by grants from the Key Project of National Natural Science Foundation of China (81230021) and the China Scholarship Council (201406160073). The Health and Retirement Study data was sponsored by the National Institute on Aging (U01AG009740) and was conducted by the University of Michigan.

## Conflict of Interest

The authors declare that the research was conducted in the absence of any commercial or financial relationships that could be construed as a potential conflict of interest.

## Publisher's Note

All claims expressed in this article are solely those of the authors and do not necessarily represent those of their affiliated organizations, or those of the publisher, the editors and the reviewers. Any product that may be evaluated in this article, or claim that may be made by its manufacturer, is not guaranteed or endorsed by the publisher.

## Abbreviations

AD: Alzheimer's Disease
ARHI: Age-related hearing impairment
AUC: Area under the curve
CI: Confidence Intervals
CVD: Cardiovascular disease
dB HL: Decibels in hearing level
GS: Gender Score
HCAP: Harmonized Cognitive Assessment Protocol
HRS: Health and Retirement Study
OR: Odds Ratio
ROC: Receiver Operator Characteristic.
