# Supplementary material for: Gender Modifies the Association of Cognition With Age-Related Hearing Impairment in the Health and Retirement Study
Source: Front Public Health. 2021 Dec 17;9:751828. doi: 10.3389/fpubh.2021.751828 (PMC8718684; doi:10.3389/fpubh.2021.751828)
Supplement: Supplementary file 1 [file Data_Sheet_1.PDF]

Supplementary Material

1 Supplementary Tables

Supplementary Table 1. Characteristics of participants between selected and excluded ones of the HRS 2016, *n* = 20,912

Characteristics	Excluded *n* = 18,000	Selected *n* = 2,912	*P* value
Median age, years (IQR)	64 (19)	64 (16)	0.0867
Sex, female	1,0771 (59.84)	1,476 (50.69)	**<0.001**
Ethnicity[a]			**<0.001**
White	11,577 (64.59)	2,247 (77.16)	
Black	4,138 (23.09)	395 (13.56)	
other	2,208 (12.32)	270 (9.27)	
Marital status[b]			**<0.001**
never married	1,580 (8.78)	72 (2.47)	
spouse absent	7,483 (41.57)	390 (13.39)	
married	8,928 (49.60)	2,450 (84.13)	
Education[c]			**<0.001**
less than high school	3,341 (18.57)	309 (10.61)	
high school or equivalence	5,817 (32.33)	936 (32.14)	
some college and above	8,837 (49.11)	1,667 (57.25)	
Current smoker[d]	2,745 (15.33)	308 (10.58)	**<0.001**
Regular drinker[e]	1,527 (8.48)	326 (11.20)	**<0.001**
Regular vigorous physical activity[f]	6,123 (34.02)	1,157 (39.73)	**<0.001**
Median CESD score (IQR)[g]	1 (2)	0 (1)	**0.0001**
≥1 negative feeling in CESD[g]	10,024 (58.78)	1,326 (45.54)	**<0.001**

Data are reported as *n* (%) unless otherwise specified. Bold values: reached the significant level of $P < 0.05$. Abbreviations: CESD = Center for Epidemiologic Studies Depression Scale, HRS = Health and Retirement Study, IQR = interquartile range. [a]*n* = 20,835, [b]*n* = 20,903, [c]*n* = 20,907, [d]*n* = 20,818, [e]*n* = 20,835, [f]*n* = 20,808, [g]*n*= 19,965

Supplementary Table 2. Selected gender-related variables from the 2016 HRS, *n* = 2,912

Variables	Description	Original HRS variables
Education	0 = lower than high school; 1 = high school; 2 = some college or above	PB014-016
Marital status	0 = never married; 1 = spouse absent/separated/divorced/widowed; 2 = married	PA020, PA023, PA026-028, PA030, PA033-035, PB055, PB058, PB061, PB065, PB068_1, PX065_R, PZ023
Smoking	0 = not smokes now; 1 = smokes now	PC117
Regular drinking	0 = drinking alcohol less than 5 days per week; 1 = drinking alcohol at least 5 days per week	PC128-129
Exercise	0 = vigorous physical activity less than once per week; 1 = vigorous physical activity at least once per week	PC223
Labor force participation	0 = not currently working for pay; 1 = currently working for pay	PJ020, PJ553
Risk attitudes	average score of assessing individuals' attitudes about risk across 5 different life domains (8) (higher score means more willing to task risks)	PLB032_1-032_5
Household income	0 = last-year household income above poverty threshold; 1 = last-year household income below poverty threshold	PN004-005, PQ431_1-431_10, PQ432_1-432_10, PQ433_1-433_10, PQ434_1-434_10, PQ435_1-435_10, PQ436_1-436_10, PQ437-440, PX056_MC, PX060_MC, PX061_MC, PX063_MC, PX065_MC, PX067_MC, PX069_MC, PX073_MC
Places of care	0 = have usual places to receive care or advice about health; 1 = have no place to receive care or advice about health	PN291

Cont'd Supplementary Table 2

Loneliness	average score of 11-item UCLA loneliness (1)	PLB019A-019K
Life satisfaction	average score of 5 items of the Satisfaction with Life Scale (2)	PLB002A-002E
Depression	sum score of 8 items of the CES-D (HRS version) (3)	PD110-117
Household tasks	average score of 5 questions of household labor (4) (higher score means less participation)	PLB005D1-005D5

Abbreviations: CES-D = Center for Epidemiologic Studies Depression, HRS = Health and Retirement Study, UCLA = The University of California, Los Angeles

Supplementary Table 3. Loadings of selected gender-related variables after factor analysis, *n* = 2,912

**Variables**	**Component 1**	**Component 2**	**Component 3**	**Component 4**	**Component 5**	**Uniqueness**
Loneliness	**0.7706**	0.0375	0.0198	0.0268	-0.0520	0.4010
Life satisfaction	**-0.7686**	0.0088	-0.0087	0.1219	0.0071	0.3942
Depression	**0.7310**	0.1559	-0.0824	-0.0305	-0.0284	0.4328
Education	-0.0449	**-0.6990**	0.2062	-0.0788	0.1407	0.4409
Household income	0.1139	**0.6390**	-0.0076	-0.2201	0.1128	0.5174
Places of care	0.0417	**0.5059**	0.3208	0.1265	-0.2901	0.5393
Smoking	0.2067	0.3662	-0.0214	-0.2631	0.3397	0.6381
Risk attitudes	0.0439	0.0308	**0.7027**	0.0853	0.0905	0.4879
Labor force participation	-0.0103	-0.2730	**0.6415**	-0.1249	-0.0464	0.4961
Exercise	-0.2845	0.0621	**0.5074**	0.0515	0.1022	0.6447
Household tasks	0.0160	0.1544	0.0366	**0.7850**	0.1367	0.3396
Marital status	-0.1427	-0.2764	-0.0318	**0.7064**	-0.0318	0.4022
Regular drinking	-0.0616	-0.0581	0.0468	0.0924	**0.8612**	0.2404

Bold items: significant loadings (> 0.40) on their corresponding component

Supplementary Table 4. Loadings of selected gender-related variables after factor analysis and the selection process, *n* = 2,912

**Variables**	**Component 1**	**Component 2**	**Component 3**	**Component 4**	**Component 5**
Loneliness	**0.7706**	0.0375	0.0198	0.0268	-0.0520
Life satisfaction	**-0.7686**	0.0088	-0.0087	0.1219	0.0071
Depression	**0.7310**	0.1559	-0.0824	-0.0305	-0.0284
Education	-0.0449	**-0.6990**	0.2062	-0.0788	0.1407
Household income	0.1139	**0.6390**	-0.0076	-0.2201	0.1128
Places of care	0.0417	**0.5059**	0.3208	0.1265	-0.2901
Risk attitudes	0.0439	0.0308	**0.7027**	0.0853	0.0905
Labor force participation	-0.0103	-0.2730	**0.6415**	-0.1249	-0.0464
Household tasks	0.0160	0.1544	0.0366	**0.7850**	0.1367
Marital status	-0.1427	-0.2764	-0.0318	**0.7064**	-0.0318
Regular drinking	-0.0616	-0.0581	0.0468	0.0924	**0.8612**

Bold items: significant loadings on their corresponding component

2 Supplementary Figures

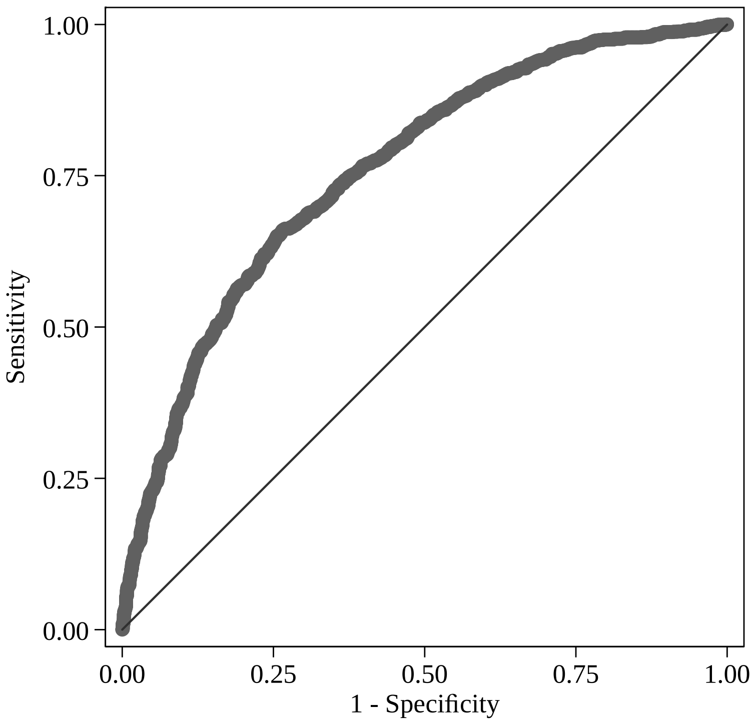

Supplementary Figure 1. ROC analysis of 2016 HRS participants for GS and sex (*n* = 2,912). GS = gender score, HRS = Health and Retirement Study, ROC = receiver operator characteristic.

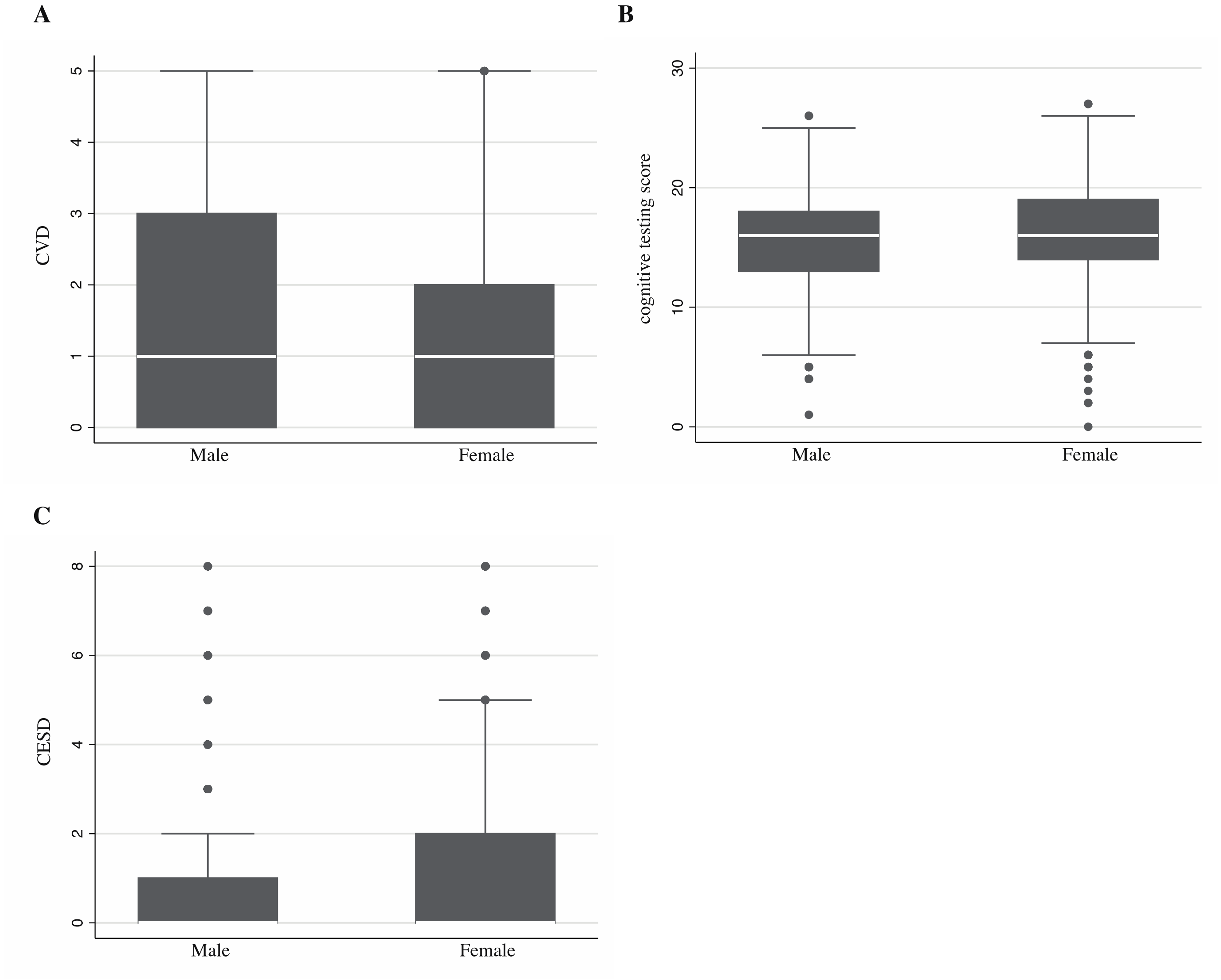

Supplementary Figure 2. Box plots of the (A) CVD, (B) cognition and (C) depression data of the 2016 HRS participants by sex (*n* = 2,912). CESD = Center for Epidemiologic Studies Depression Scale, CVD = cardiovascular disease, HRS = Health and Retirement Study.

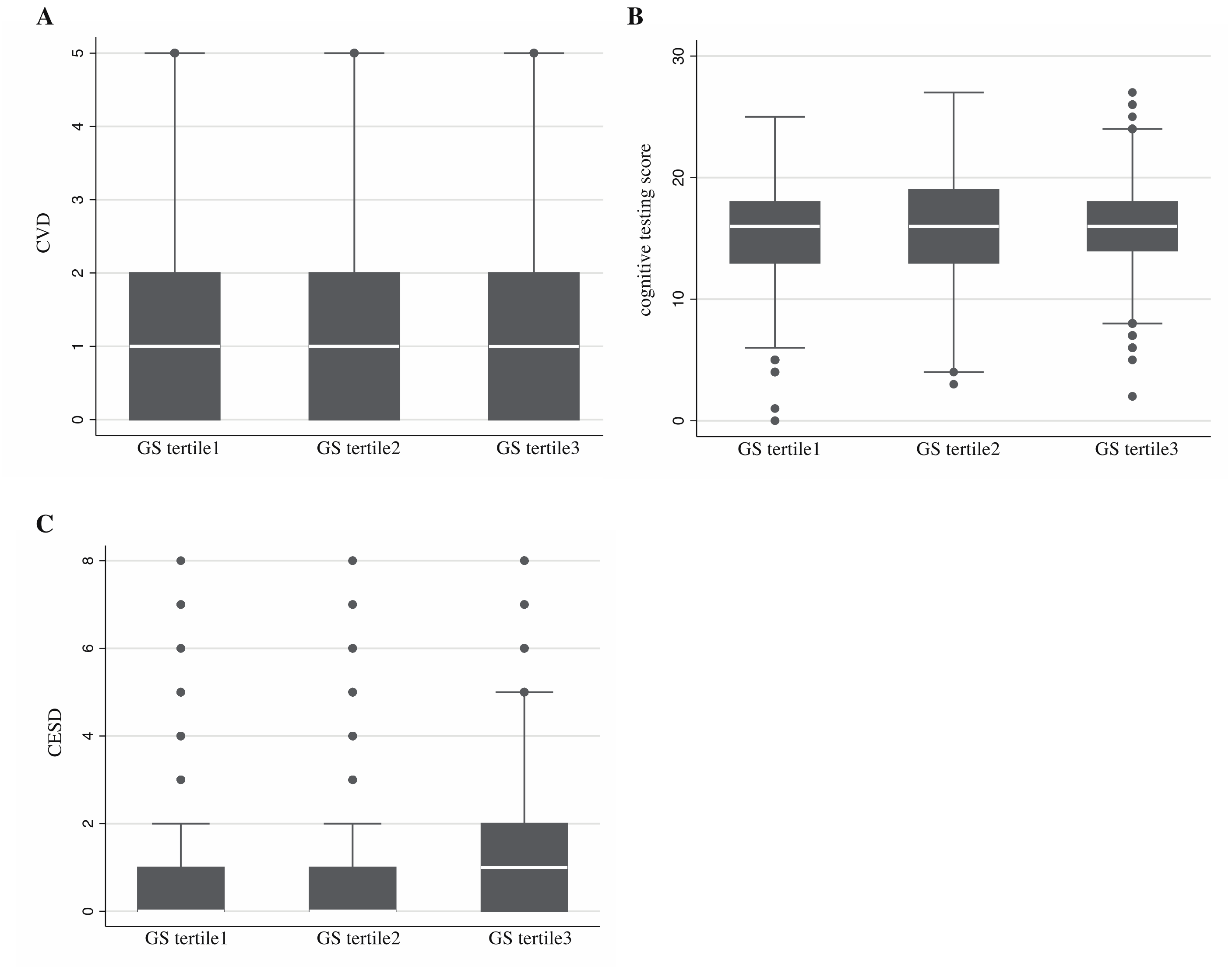

Supplementary Figure 3. Box plots of the (A) CVD, (B) cognition and (C) depression data of the 2016 HRS participants by GS tertile (*n* = 2,912). CESD = Center for Epidemiologic Studies Depression Scale, CVD = cardiovascular disease, GS = gender score, HRS = Health and Retirement Study.

REFERENCES

1. Russell DW. UCLA Loneliness Scale (Version 3): Reliability, Validity, and Factor Structure. *J Pers Assess* (1996) 66:20-40. doi: 10.1207/s15327752jpa6601_2

2. Diener E, Emmons RA, Larsen RJ, Griffin S. The Satisfaction with Life Scale. *J Pers Assess* (1985) 49:71-5.

3. Steffick DE. Documentation of Affective Functioning Measures in the Health and Retirement Study Ann Arbor, MI: Institute for Social Research, University of Michigan (2000). https://hrs.isr.umich.edu/publications/biblio/5411 [Accessed March 30, 2021].

4. Kamo Y. “He Said, She Said”: Assessing Discrepancies in Husbands' and Wives' Reports on the Division of Household Labor. *Soc Sci Res* (2000) 29:459-76. doi: 10.1006/ssre.2000.0674
